# Next-Generation Sequencing Reveals Significant Bacterial Diversity of Botrytized Wine

**DOI:** 10.1371/journal.pone.0036357

**Published:** 2012-05-01

**Authors:** Nicholas A. Bokulich, C. M. Lucy Joseph, Greg Allen, Andrew K. Benson, David A. Mills

**Affiliations:** 1 Department of Viticulture and Enology, University of California Davis, Davis, California, United States of America; 2 Department of Food Science and Technology, University of California Davis, Davis, California, United States of America; 3 Dolce Winery, Oakville, California, United States of America; 4 Department of Food Science, University of Nebraska, Lincoln, Nebraska, United States of America; University of Vienna, Austria

## Abstract

While wine fermentation has long been known to involve complex microbial communities, the composition and role of bacteria other than a select set of lactic acid bacteria (LAB) has often been assumed either negligible or detrimental. This study served as a pilot study for using barcoded amplicon next-generation sequencing to profile bacterial community structure in wines and grape musts, comparing the taxonomic depth achieved by sequencing two different domains of prokaryotic 16S rDNA (V4 and V5). This study was designed to serve two goals: 1) to empirically determine the most taxonomically informative 16S rDNA target region for barcoded amplicon sequencing of wine, comparing V4 and V5 domains of bacterial 16S rDNA to terminal restriction fragment length polymorphism (TRFLP) of LAB communities; and 2) to explore the bacterial communities of wine fermentation to better understand the biodiversity of wine at a depth previously unattainable using other techniques. Analysis of amplicons from the V4 and V5 provided similar views of the bacterial communities of botrytized wine fermentations, revealing a broad diversity of low-abundance taxa not traditionally associated with wine, as well as atypical LAB communities initially detected by TRFLP. The V4 domain was determined as the more suitable read for wine ecology studies, as it provided greater taxonomic depth for profiling LAB communities. In addition, targeted enrichment was used to isolate two species of *Alphaproteobacteria* from a finished fermentation. Significant differences in diversity between inoculated and uninoculated samples suggest that *Saccharomyces* inoculation exerts selective pressure on bacterial diversity in these fermentations, most notably suppressing abundance of acetic acid bacteria. These results determine the bacterial diversity of botrytized wines to be far higher than previously realized, providing further insight into the fermentation dynamics of these wines, and demonstrate the utility of next-generation sequencing for wine ecology studies.

## Introduction

The past decade has seen a phenomenal leap forward in understanding the microbial ecology of wine fermentations, as molecular profiling methods have been adopted to further explore microbial systems inhabiting grapes, barrels, wineries, and wines [1]. Prospecting the biodiversity of wine fermentations expands our understanding of fermentation control and of problem fermentations, enables discovery of novel starter cultures, and provides a framework for the “normal" microbiota of wine fermentation (as well as identifying point sources of microbial contamination) as diagnostic and profiling tools move from academia into the industrial arena. The approach also portends the discovery of links between microbial populations and flavor development, allowing keen insights into the origins of organoleptic properties.

So-called next-generation sequencing (NGS) technologies have ushered in a new era of biodiversity surveillance, enabling high-throughput analysis of complex microbial communities via short amplicons, typically hypervariable domains of prokaryotic 16S rDNA. Given the scale of sequencing reactions possible in a single run of most NGS platforms, hundreds to thousands of samples may be multiplexed using short DNA sequence “barcodes" [2], providing adequate sequencing depth in each sample to characterize the top 99.99% of the microbiota. This has facilitated comparative ecological analysis on a large scale and—with sensitivity well beyond that of first-generation profiling technologies—provides relatively quantitative comparisons of microbial communities across ecosystems at depths previously unattainable [3].

Among wine fermentations, those produced from botrytized grapes are known to involve an unusually high diversity of yeasts [4], [5], [6] and acetic acid bacteria [7]. These grapes are infected by the mold *Botrytis cinerea* during extended ripening time prior to harvest, dehydrating the grape berries, which leads to elevated sugar concentrations in the must [8]. One such wine with a long history of study is Dolce (Oakville, CA), produced from botrytized Sauvignon Blanc and Semillon grapes. Earlier vintages of this wine were first studied using DGGE, revealing the involvement of diverse yeast communities, including the fructophilic yeast *Candida zemplinina* [4], [6]. Using TRFLP, the 2008 and 2009 vintages demonstrated a similar set of yeasts, including *C. zemplinina* and other yeasts not typically isolated from wine fermentations, particularly batches not inoculated with *Saccharomyces cerevisiae* [9]. The elevated sugar concentrations and decreased fermentation temperature common to most botrytized wine fermentations appear to enrich for these unusual yeast communities, particularly *C. zemplinina*, which was originally isolated from Dolce [4] and botrytized Tokaj wines [10]. Little is known about the bacterial communities involved in botrytized wines or whether a similarly selective environment for technologically promising species (e.g., high ethanol tolerance) is formed by the high-sugar, low-temperature conditions of fermentation. Given the wealth of yeasts consistently detected in Dolce fermentations as well as preliminary denaturing gradient gel electrophoresis (DGGE) data [11], this fermentation was expected to involve similarly diverse bacterial communities, so was selected for this study.

This work describes the first such look into the rare bacterial taxa of wine, using barcoded amplicon sequencing (BAS) as a tool for biodiversity surveillance. As a primary goal of this work, we tested two different hypervariable domains (V4 and V5) of the bacterial 16S rRNA gene for their suitability for profiling the bacterial communities in wine fermentations via sequencing on the Illumina GAIIx. V4 and V5 16S rDNA amplicons provided slightly different views of the fermentation communities, and different advantages, but the V4 provides greater taxonomic depth for profiling lactic acid bacteria (LAB) communities. In addition, sequence analysis revealed the presence of several taxa not traditionally associated with wine fermentation, leading to targeted culturing of two such bacteria from this wine.

## Methods

### Sample Collection

**Figure 1:**
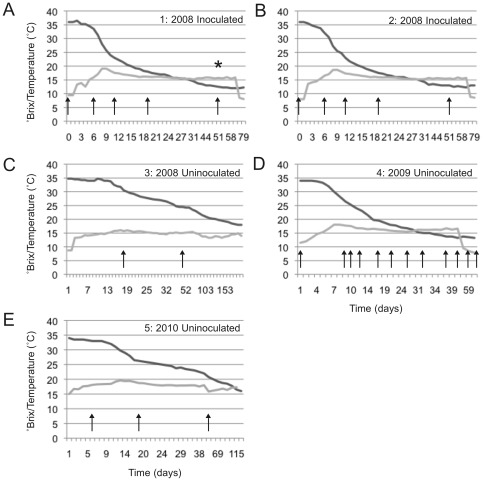
Fermentation rate and temperature of Dolce fermentations. Panel A: Batch 1, 2008 inoculated; Panel B: Batch 2, 2008 inoculated; Panel C: Batch 3, 2008 uninoculated; Panel D: Batch 4, 2009 uninoculated; Panel E: Batch 5, 2010 uninoculated. Dark grey = °Brix, Light grey = Temperature (°C). Arrows represent sampling times. * Indicates sample used for culture-dependent analysis.

Samples of botrytized wine fermentations (Dolce Winery, Oakville, CA) were collected aseptically from three separate vintages (2008, 2009, and 2010), frozen at −20°C, transported on ice, and stored at −80°C until processing. Samples from 2008 represented three separate batches, two inoculated with *Saccharomyces cerevisiae* (batches 1, 2) and one uninoculated (batch 3), as well as two press-pan samples collected following juice pressing. Samples from 2009 and 2010 represented one uninoculated batch each (batches 4 and 5, respectively). Fermentation rate curves and sampling times are presented in Figure 1. Fermentations were conducted at ambient temperature without temperature control.

### DNA Extraction

Samples were processed according to the modified protocol of Martinez and coworkers [12]. Samples were centrifuged at 4,000× g for 10 min and decanted. The resulting cell pellet was resuspended in residual supernatant, 100 µL were removed and washed 3 times by suspension in 1 mL ice-cold PBS, centrifugation at 8,000× g (5 min), and the supernatant discarded. The pellet was then suspended in 200 µL DNeasy lysis buffer (20 mM Tris-Cl [pH 8.0], 2 mM Sodium EDTA, 1.2% Triton X-100) supplemented with 40 mg/mL lysozyme and incubated at 37°C for 30 min. From this point, the extraction proceeded following the protocol of the Qiagen Fecal DNA Extraction Kit (Qiagen, Valencia, CA), with the addition of a bead beater cell lysis step of 2 min at maximum speed following addition of “buffer ASL" using a FastPrep-24 bead beater (MP Bio, Solon, OH). DNA extracts were stored at −20°C until further analysis.

### Library Construction

For amplification of the V4 domain of bacterial 16S rDNA, we used primers F515 (5′-CACGGTCGKCGGCGCCATT-3′) and R806 (5′-GGACTACHVGGGTWTCTAAT-3′) [3], both modified to contain an Illumina adapter region for sequencing on the Illumina GAIIx platform and, on the forward primer, an 8 bp Hamming error-correcting barcode to enable sample multiplexing [2]. A list of V4 primers and barcodes used is presented in Table S1. PCR reactions contained 5–100 ng DNA template, 1× GoTaq Green Master Mix (Promega, Madison, WI), 1 mM MgCl2, and 2 pmol of each primer. Reaction conditions consisted of an initial 94°C for 3 min followed by 35 cycles of 94°C for 45 sec, 50°C for 60 sec, and 72°C for 90 sec, and a final extension of 72°C for 10 min. All samples were amplified in triplicate and combined prior to purification. Amplicons were purified using the Qiaquick 96 kit (Qiagen), quantified using PicoGreen dsDNA reagent (Invitrogen, Grand Island, NY) on a 96-well plate reader, mixed at equimolar concentrations, and gel purified using the Qiaquick gel extraction kit (Qiagen) all according to respective manufacturers' instructions.

For amplification of the V5 domain of bacterial 16S rDNA, primers 786F (5′-GATTAGATACCCTGGTAG-3′) and 926R (5′-CCGTCAATTCMTTTGAGTTT-3′) were used [13], with the forward primer modified to contain a 6 bp non-error-correcting barcode at the 5′ terminus (a list of the barcodes used is presented in Table S2). Amplicons were quantified using Picogreen dsDNA reagent (Invitrogen), mixed at an equimolar concentration, and purified using Qiaquick spin kit (Qiagen). The V4 library was prepared from pooled amplicons by ligation of the Illumina adapters using the Illumina paired-end DNA sample preparation kit.

Purified libraries were submitted to the UC Davis Genome Center DNA Technologies Core for cluster generation and 150 bp paired-end sequencing on the Illumina GAIIx platform. V4 and V5 samples were submitted in two separate runs, each containing barcoded samples from another, unrelated study. Image analysis, base calling, and error estimation were performed using CASAVA 1.7.

### Data Analysis

Raw Illumina fastq files were demultiplexed, quality-filtered, and analyzed using QIIME [14]. The 150-bp reads were truncated at any site of more than three sequential bases receiving a quality score <1e-5, and any read containing ambiguous base calls or barcode/primer errors were discarded, as were truncated reads of <75 bp and reads with <60 consecutive high-quality base calls. Operational Taxonomic Units (OTUs) were assigned using the QIIME implementation of UCLUST [15], with a threshold of 97% pairwise identity, and representative sequences from each OTU selected for taxonomy assignment. OTUs were classified taxonomically using a QIIME-based wrapper of the Ribosomal Database Project classifier program [16] against the RDP core set [17], [18], using a 0.80 confidence threshold for taxonomic assignment. Unassigned sequences (including unidentifiable bacteria), plastid sequences, and any OTU comprising less than 0.01% of total sequences for each run were removed prior to further analysis.

Beta diversity estimates were calculated within QIIME using weighted Unifrac distances [19] between samples subsampled 20 times, with replacement, at a depth of 100 sequences per sample. From these estimates, jackknifed principal coordinates (PC) were computed to compress dimensionality into two-dimensional principal coordinate analysis (PCoA) plots. QIIME was also used to calculate alpha diversity on rarefied OTU tables to assess sampling depth coverage using observed species and phylogenetic diversity (PD) [20] metrics, as well as Martin's P test [21], G test of independence, and ANOVA between all sample pairs to test significant differences in beta diversity.

### LAB-TRFLP

Lactic acid bacteria (LAB)-specific TRFLP (LAB-TRFLP) was performed as described previously [22]. Briefly, samples were amplified by PCR in 50-µL reactions containing 5–100 ng of DNA template, 25 µL 2× GoTaq Green Master Mix (Promega), 1 mM MgCl_2_, and 2 pmol of each primer (NLAB2F, 5′-[HEX]-GGCGGCGTGCCTAATACATGCAAGT; and WLAB1R, 5′-TCGCTTTACGCCCAATAAATCCGGA-3′). The forward primer was labeled with hexachlorofluorescin (HEX). Each PCR was performed in triplicate and the products combined prior to purification. The PCR conditions consisted of an initial denaturation at 95°C for 5 min, followed by 30 cycles of denaturation at 95°C for 45 sec, annealing at 66°C for 30 sec, and extension at 72°C for 45 sec, and with a final extension at 72°C for 5 min. Amplicons were digested using Hpy188I and MseI following the manufacturers' instructions for each enzyme. The digested DNA was submitted to the UC Davis College of Biological Sciences Sequencing Facility for fragment analysis. Traces were visualized using the program Peak Scanner v1.0 (Applied Biosystems) using a baseline detection value of 10 fluorescence units. Peak filtration and clustering were performed with R software using the IBEST script suite [23]. OTU picking was based on an *in silico* digest database generated by the virtual digest tool from MiCA [24] of good-quality 16S rRNA gene sequences compiled by the Ribosomal Database Project Release 10 [17], [18], allowing up to 3 nucleotide mismatches within 15 bp of the 5′ terminus of the forward primer.

### Culture-dependent Analysis

Based on sequence analysis, a targeted approach was used to culture sphingomonads detected in late-fermentation Dolce samples. A sample from day 51 of fermentation, batch 3, 2008 vintage (Figure 1), was enriched in sphingomonas broth [25] supplemented with 5% ethanol and erythromycin under microaerobic conditions. Isolates were identified by colony PCR with the primers 63F (5′- CAGGCCTAACACATGCAAGTC -3′) and 1387R (5′- GGGCGGWGTGTACAAGGC -3′) [26] and submitted to the UC Davis College of Biological Sciences Sequencing Facility for sequencing. Isolates were deposited in the UC Davis Viticulture and Enology Culture Collection: *Methylobacterium populi* (UCD404) and *Sphingomonas pseudosanguinis* (UCD405).

## Results

While wine fermentation has long been known to involve complex microbial communities, the composition and role of bacteria other than a select set of malolactic LAB has often been assumed negligible or else detrimental. We chose to study Dolce wine fermentation because, based on previous studies of the same vintages, these fermentations contain complex, unusual yeast communities [9], and previous DGGE surveys of the Dolce fermentation [11] predicted that the bacterial communities would exhibit similar diversity.

Paired-end sequencing of V4 and V5 16S rDNA amplicons was performed in two separate runs on the Illumina GAIIx. The 5′ and 3′ reads were analyzed separately, as QIIME [14] currently does not handle paired-end data. Additionally, others have shown that concatenating paired-end reads does not necessarily improve taxonomic depth and phylogenetic analyses [27], so we handled 5′ and 3′ reads independently, with the purpose of comparing their efficacy as single-direction reads. Raw read counts and quality filtration statistics for each run are presented in Table S3. Alpha rarefaction plots of observed species (Figure S1, left) were constructed to determine that adequate sequence coverage was obtained to reliably describe the full diversity present in these samples. Samples exhibiting largely inadequate sequencing depth, as indicated by a non-asymptotic rarefaction curve, were removed prior to further analyses. Additionally, the PD metric (Figure S1, right), which measures the complete phylogenetic distance represented within a community [20], demonstrated an apparently greater level of phylogenetic diversity in V4 reads compared to V5 reads.

**Figure 2:**
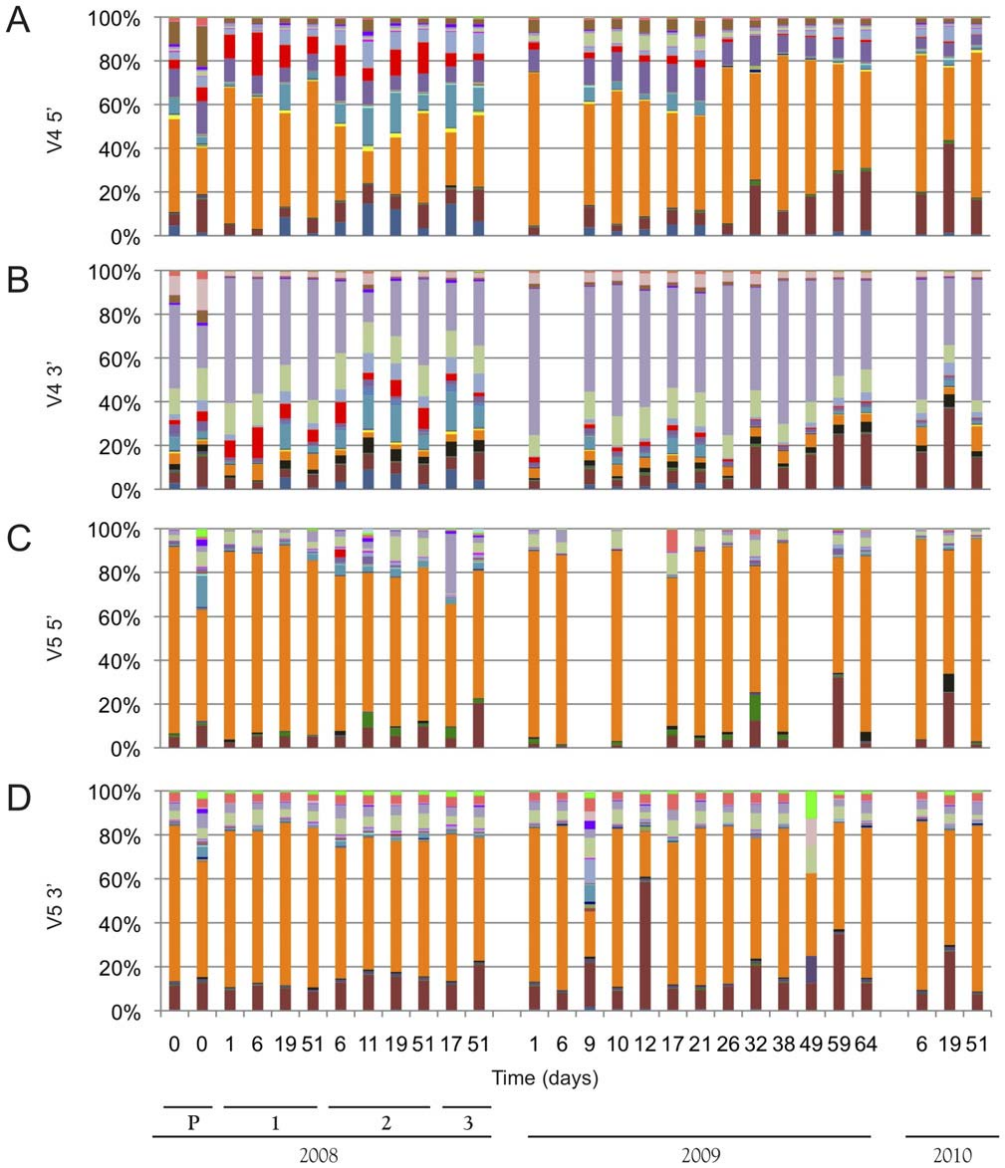
Bacterial community structure determined by sequencing of the V4 (Panels A,B) and V5 (Panels C,D) domain of 16S rRNA. Panel A: V4 5′. Panel B: V4 3′. Panel C: V5 5′. Panel D: V5 3′. Labeled bars indicate batch numbers (P, 1–3) and vintage (2008, 2009, 2010). *y*-axis represents relative OTU abundance. P indicates 2008 press-pan samples. Missing samples were removed due to inadequate sequence coverage. For color key, see Figure S2.

BAS of Dolce fermentation samples identified a range of minor microbiota, many of which, to our knowledge, have not been reported in wine previously (Figure 2, Figure S2). In general, bacterial community structure exhibited little change across the fermentation, except for a gradual reduction of *Proteobacteria* and increase of *Firmicutes* over time. In both vintages, *Rhodospirillales* (predominantly *Acetobacter*, *Gluconobacter*, and *Gluconoacetobacter*) were the most dominant bacteria detected, with secondary populations of *Lactobacillales*. Fluctuating, minor populations (some as high as 10% of total sequences detected but typically <1%) of *Chryseobacterium* (*Bacteroidetes*), *Methylobacterium* and *Sphingomonas* (*Alphaproteobacteria*), *Arcobacter* (*Eplisoniproteobacteria*), *Naxibacter* and *Ralstonia* (*Betaproteobacteria*), *Frigoribacterium* (*Actinobacteria*), and *Pseudomonas*, *Zymobacter*, and *Acinetobacter* (*Gammaproteobacteria*) were also observed at different times, particularly in the 2009 vintage. For a complete list of genera detected, see Tables S4, S5, S6, and S7.

Sequencing of both the V4 and V5 regions provided similar views of community structure in these wines, albeit with different degrees of evenness. The V5 region displayed a global dominance of *Acetobacteriaceae*, while V4 data suggest that *Firmicutes* and other *Proteobacteria* represent a larger relative portion of the microbiota in these samples. Both resulted in similar taxonomic assignments, with slight differences at the genus level. The V4, for example, produced a higher number of genus-level assignments meeting threshold criteria for select *Proteobacteria* (e.g., *Enterobacteriaceae* and *Burkholderiaceae*) and *Firmicutes*, particularly *Lactobacillaceae.* The *Lactobacillaceae* were particularly disparate in assignment as the V5 data had only taxa assigned as “other *Lactobacillales*". Taxonomic assignments from V5 sequences, however, were less sensitive to truncation, such that sequences truncated to <100 nt due to low-quality base calls were still assigned to order- and family-level taxonomic ranks, whereas truncated V4 sequences of equivalent length were assignable only at the phylum and class levels. For both regions, the 5′ read was slightly more taxonomically informative than the 3′ read (as these were analyzed separately in QIIME) but generally revealed the same community structure. The V4 3′ reads, in particular, selectively achieved shallower taxonomic resolution, most readily observed as the assignment of the dominant OTU as *Proteobacteria* (as opposed to *Gluconobacter* [*Rhodospirillales*] by the V4 5′ and V5 reads; Figure 2, Figure S2), but with assignment of LAB comparable to that of other reads.

**Figure 3:**
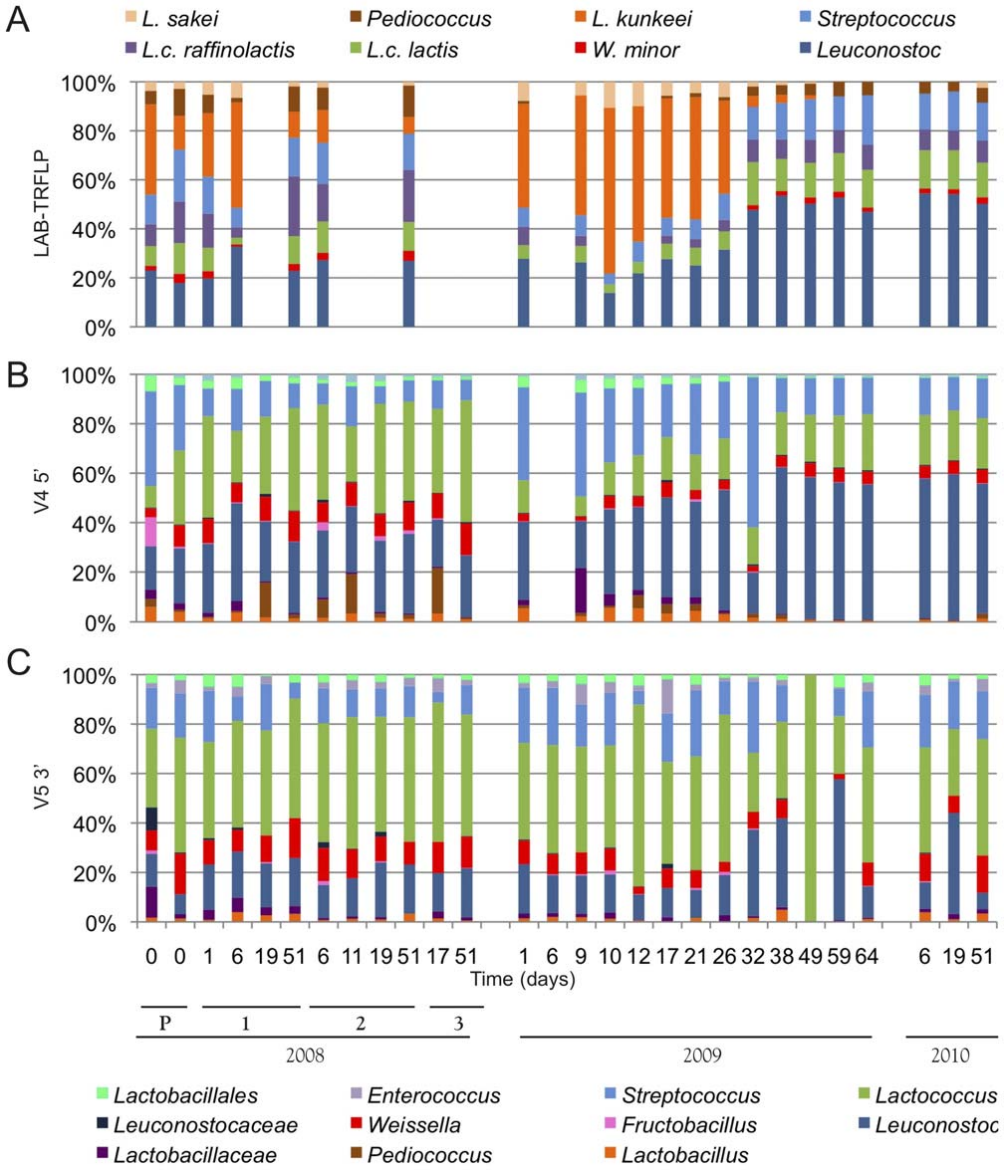
*Lactobacillales* community of Dolce fermentation revealed by LAB-TRFLP (Panel A), V4 5′ read (Panel B) and V5 3′ read (Panel C). Top, taxonomy key for LAB-TRFLP. Bottom, taxonomy key for V4 and V5 sequencing. Labeled bars indicate batch numbers (1–3) and vintage (2008 and 2009). *y*-axis represents relative OTU abundance. Missing samples were unable to amplify by LAB-TRFLP. P indicates 2008 press-pan samples.

Most OTUs could be resolved to family-level, and many to genus, in spite of the short read length employed (150 bp). However, many *Lactobacillales* could not be further discriminated. As this is the most important bacterial order to wine fermentation (both for spoilage potential and malolactic activity), sequencing data were augmented by and compared to LAB-TRFLP [22], which can identify most LAB to species (Figure 3). In both vintages, V5 sequencing identified *Lactococcus* as the most dominant genus, with secondary populations of *Leuconostoc* and *Weissella* and minor populations of *Streptococcus* and *Fructobacillus* (Figure 3B). This was roughly mirrored by V4 sequencing as a dominance by *Leuconostoc* and *Lactobacillales* with a significant population of *Lactococcus* (Figure 3C). LAB-TRFLP presents a very different picture of the fermentation, particularly from the V5 reads (Figure 3A). *Lactobacillus kunkeei* and *Leuconostoc* spp. dominated the early and late fermentations, respectively, while *Lactococcus lactis*, *Lactococcus raffinolactis*, *Weissella minor*, *Streptococcus*, *Lactobacillus sakei*, and *Pediococcus* were all detected as minor populations. The V4 reads, exhibiting increased relative abundances of *Lactobacillaceae* and *Pediococcus* and decreased abundance of *Lactococcus*, were more comparable to LAB-TRFLP than the V5 reads.

**Figure 4:**
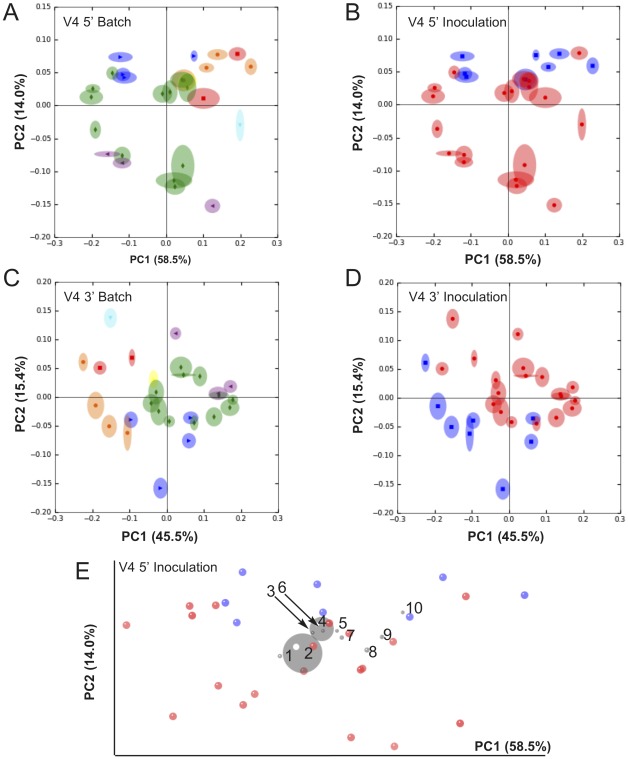
Inoculation and batch direct bacterial diversity of Dolce fermentations. Jackknifed Weighted UniFrac PCoA of V4 sequences categorized by batch number (A,C) and inoculation (B,D,E). Panel A–B: V4 5′ read weighted UniFrac. Panel C–D: V4 3′ read weighted UniFrac. Panel E: PCoA biplot displaying OTU (as loadings, grey orbs) correlation to samples categorized by inoculation; OTU labels correspond to ID numbers in Table 1. Color codes for batch-categorization (A,C): Blue = batch 1, 2008 inoculated; Orange = batch 2, 2008 inoculated; Red = batch 3, 2008 uninoculated; Green = batch 4, 2009 uninoculated; Purple = batch 5, 2010 uninoculated; Yellow = 2008 press-pan sample 1; Cyan = 2008 press-pan sample 2. Color codes for inoculation-categorization (B,D,E): Blue = inoculated; Red = uninoculated.

In order to view relationships among samples based on differences in phylogenetic diversity, principle coordinates (PC) were calculated from jackknifed UniFrac distances [19] between samples and used to construct three-dimensional principal coordinate analysis (PCoA) plots (Figure 4). Samples cluster by batch based on weighted UniFrac distance (Figure 4A,C), with clear separation of inoculated and uninoculated samples (Figure 4 B,D). Samples did not cluster based on age (weeks of fermentation), implying that the overall phylogenetic diversity changes little during the course of the fermentation. Cluster separation was less distinct based on V5 sequence data (data not shown). A P test [21] confirmed significant differences in genetic diversity between each batch (Bonferroni-corrected *p*<0.001) and between inoculated/uninoculated batches (Bonferroni-corrected *p*<0.001) with 1000 Monte Carlo iterations. Based on this significant result, we used ANOVA to test category-specific differences in abundance among OTUs assigned to the V4 5′ sequences. Ten OTUs demonstrate significant differences (false discovery rate-corrected *p*<0.05) between inoculated and uninoculated groups (Table 1). A G test of independence verified that these differences were not significantly related to presence/absence of any OTU between groups (false discovery rate-corrected *p*>0.05). To visualize relationships among these significant OTUs and sample types, we constructed a PCoA biplot plotting significant OTUs (as loadings) in relation to samples (Figure 4E). This plot was constructed from the weighted UniFrac PCoA of V4 5′ reads (Figure 4B), but OTUs are given coordinates in addition to samples in order to show how OTUs correlate with samples along the principle coordinates. OTU coordinates are indicated by grey orbs with size as a function of relative abundance, and labeled according to ID in Table 1. Most of the OTUs appear to associate more strongly with uninoculated samples, especially *Gluconobacter*. Two OTUs, *Zymobacter* and *Dyella*, appear to be more associated with inoculated samples.

**Table 1:** ANOVA Significance of Inoculation-based Differences in Bacterial Taxa.

ID^a^	FDR-*p* b	Taxon
1	0.03857	*Proteobacteria; Alphaproteobacteria; Rhodospirillales; Acetobacteraceae; Gluconobacter*
2	0.03054	*Proteobacteria; Alphaproteobacteria; Rhodospirillales; Acetobacteraceae; Gluconobacter*
3	0.01259	*Proteobacteria; Alphaproteobacteria; Rhodospirillales; Acetobacteraceae; Gluconobacter*
4	0.00015	*Proteobacteria; Gammaproteobacteria; Oceanospirillales; Halomonadaceae; Zymobacter*
5	0.00001	*Proteobacteria*
6	0.00523	*Proteobacteria*
7	0.00189	*Actinobacteria; Actinobacteria; Bifidobacteriales; Bifidobacteriaceae; Bifidobacterium*
8	0.02991	*Proteobacteria; Betaproteobacteria; Burkholderiales; Comamonadaceae*
9	0.04960	*Proteobacteria; Alphaproteobacteria; Sphingomonadales; Sphingomonadaceae; Sphingomonas*
10	0.01549	*Proteobacteria; Gammaproteobacteria; Xanthomonadales; Xanthomonadaceae; Dyella*

a ID indicates OTU placement in PCoA biplot (Figure 4E).

b FDR-*p*: False discovery rate-corrected *p*-value.

Considering the high bacterial diversity exhibited in these samples based on BAS data, including species not previously found in wine, it was questioned whether some sequences represent residual DNA or spores from nonviable, plant-associated bacteria, or even sequencing artifact. In particular, the persistence of *Sphingomonas* and *Alphaproteobacteria* other than *Acetobacteriaceae* was surprising. Thus, an attempt was made to culture these low-abundance species from a finished fermentation using enrichment culture. To target this genus, we used sphingomonas broth containing 5% ethanol and erythromycin under microaerobic conditions to select for species competent under alcoholic conditions and to prevent growth of aerobic bacteria, primarily *Acetobacteriaceae* and spore-forming *Bacillaceae*. Under these conditions, two isolates were obtained representing the two prevailing colony morphotypes observed on sphingomonas agar plates. The closest matches identified by 16S rDNA sequencing were *Methylobacterium populi* and *Sphingomonas pseudosanguinis*. Surprisingly, neither of these isolates was capable of growth at wine-like conditions when cultured in high-ethanol, low-pH media (data not shown).

## Discussion

The availability of cost-effective NGS methods has fundamentally enhanced our understanding of microbially dominated ecosystems, and our study here now adds further insight into a new ecosystem, wine fermentation, that has yet to be explored with this technology. With the unprecedented depth of sequencing available across each of our samples, we identified a surprisingly diverse set of bacterial clades not traditionally associated with wine fermentations. Ecologically, the most logical source for the majority of these bacteria is the *Vitis vinifera* phyllosphere, wherein *Methylobacterium* and *Sphingomonas* (among a number of bacteria detected in this study) have been detected previously using pyrosequencing of grape surfaces [28]. Other genera detected include members that are potential plant pathogens (e.g., some species of *Pseudomonas*, *Ralstonia*), nitrogen-fixing bacteria (e.g., some *Oxalobacteraceae*) and soil bacteria (e.g., some *Acinetobacter*, *Microbacteriaceae*). While an ambitious effort was not made to isolate members of all of these groups, the isolation of *Methylobacterium* and *Sphingomonas* after 51 days of fermentation illustrates that at least some of these OTUs represent viable cultures surviving well into the wine fermentation. Our discovery of these organisms begs the question of whether they are truly metabolically active and capable of affecting organoleptic properties of the wine in any measureable way. The relative abundances of these sequences remained relatively constant across the fermentations, implying that the bacteria are likely surviving in a dormant state rather than growing and/or metabolically active. Nevertheless, as standards for quality filtration have yet to be established for Illumina-platform sequence data [3], as they have been for pyrosequencing [29], [30], it is important to verify that the rare OTUs detected by BAS are neither artifact nor phantoms generated by sequencing error.

As BAS targets such a short segment of the 16S rDNA, care must be taken to select a region that is both taxonomically informative and exhibits broad coverage of all bacterial phyla. Suitability will vary depending upon sample type and associated microbiota [31], yet greater taxonomic depth will not necessarily mean “better," as different regions and primer sets tend to have different types of bias toward amplification of certain taxa and therefore offer incomplete and quantitatively skewed views of the microbiome as well as differential susceptibilities to error/chimera formation [31]. In our study, both the V4 and V5 provided comparable representations of community structure at higher taxonomic levels, with only slight differences in lower taxa. V4 5′ reads revealed broader phylogenetic diversity overall, deeper resolution of certain *Proteobacteria* (e.g., *Enterobacteriaceae* and *Burkholderiaceae*), and yielded LAB community profiles more similar to LAB-TRFLP than the V5 reads. V4 3′ reads demonstrated shallower taxonomic assignment, which is explained by the fact that the binding site for this primer is located deeper in the conserved region separating the V4 and V5 domains. Therefore, less taxonomically useful, hypervariable sequence is covered by this read, leading to relatively poor taxonomic assignment. The V5 retained more taxonomic information in heavily truncated (<100 nt) sequences compared to V4, but the V4 appears more favorable to fermentation studies based on its closer comparison to LAB-TRFLP profiles. This corroborates the results of others, who found that the V4 was a more taxonomically informative region for BAS [31], [32] and for accurate assignment using the RDP naïve Bayesian classification algorithm [16].

The greater diversity of LAB revealed by V4 sequences compared to V5 sequences (including detection of *Lactobacillaceae*) may indicate either greater phylogenetic divergence among LAB in the V4 region, or else primer bias exhibited by each primer set toward different clades. The V4 reads, with richer diversity of *Lactobacillaceae*, parallel the profile given by LAB-TRFLP much more closely than does the V5, and thus may portray a more accurate view of the LAB community. The short reads provided by NGS tools, compounded with PCR and sequencing errors, may lead to misidentification of some OTUs as closely related taxa [29]. Additionally, until quality filtration standards are developed for Illumina amplicon sequencing data [3], fairly stringent filtration parameters should be set during demultiplexing and OTU filtration steps in order to avoid overestimates of diversity, leading to the loss of sensitivity for detecting lower abundance OTUs. As LAB-TRFLP targets *Lactobacillales* specifically (limiting amplification bias to within the order) and as TRFLP-based taxonomic assignment is less sensitive to polymerase errors than sequence-based assignment (as assignment is only affected when the restriction site is altered), it is our opinion that LAB-TRFLP is more accurate for genus- and species-level taxonomic classification of LAB. This highlights the utility of such clade-specific techniques for resolving important groups (e.g., LAB in food fermentations) to lower taxonomic levels, as well as confirming the presence of low-abundance OTUs, in conjunction with NGS tools.

This study is directly significant to the winemaking community, in revealing that botrytized wine fermentations (as represented by Dolce) involve complex bacterial communities that appear to be atypical for most wine fermentations, albeit by comparison to studies employing older, less sensitive techniques (this being the first use of NGS in wine) in botrytized laboratory-scale fermentations [33] and non-botrytized wines [34], [35], [36]. Aside from the rare bacterial taxa discussed above, which have not been previously described in wine, acetic acid bacteria were particularly abundant in these fermentations, consistent with the findings of others that acetic acid bacteria exhibit significantly greater abundance in botrytized wine fermentations compared to unaffected wines [7]. The LAB community of Dolce was also abnormal, comprising primarily *Leuconostoc* and *Lactococcus* in addition to the more typical *Lactobacillus* and *Pediococcus*; notably, *Oenococcus* was entirely absent. We previously analyzed the yeast communities in these same vintages of Dolce using TRFLP [22], revealing a similarly diverse and unusual community. The atypical microbial communities of botrytized wines are most likely enriched by the physiological impact of *Botrytis* colonization on the grape berry [5], [37], and may explain the sluggish fermentations typically observed with botrytized musts [38], which purposely arrest prior to full attenuation, resulting in the sweet dessert wines typified by Sauternes [8]. As this is—to our knowledge—the first use of NGS sequencing to deeply profile the bacterial communities of botrytized wine, it is unclear whether these observations are unique to Dolce or whether this can be attributed to a larger trend. The bacterial communities of botrytized and other infected/damaged grapes should be more comprehensively studied across a broad geographic range to better define what impact these have on the fermentation and stability of wine. The increased sensitivity and throughput of NGS tools will facilitate such large-scale surveillance.

Beta-diversity, particularly phylogeny-based metrics, represent one downstream output generated from NGS data with high value to wine studies. Corresponding to phylogenetic differences uncovered by BAS, UniFrac PCoA demonstrated different cluster affinities, with V4 reads affording better separation of sample clusters. Jackknifed weighted UniFrac distance revealed batch-dependent clustering of samples, indicating that samples differed by both phylogenetic diversity and species abundance. In particular, batches inoculated with *Saccharomyces cerevisiae* (1 and 2) co-clustered tightly while uninoculated batches (3, 4, 5, press pan) formed separate, more diffuse clusters. This separation was confirmed to be significant by P tests between samples (*p*<0.001). Thus, inoculation appears to exert selective pressure on the bacterial community. This is not a surprising result, as inoculation increased rate of fermentation and thus temperature in most of the Dolce fermentations—these were not temperature-controlled fermentations, so temperature was a function of ambient temperature and microbial metabolism (Figure 1). Elevated ethanol concentration and temperature would be expected to impact diversity [4], [39], and we found a similar effect of inoculation on the yeast diversity in these same fermentations previously [9]. Eight OTUs were significantly suppressed in inoculated samples, including *Gluconobacter,* unidentified *Proteobacteria*, and *Sphingomonas* (Table 1), while *Zymobacter* and *Dyella* were enhanced in inoculated samples. *Gluconobacter* in particular can cause problem fermentations through production of acetic acid—both inhibiting *Saccharomyces* and spoiling the wine [38]—but the other bacteria on this list have not previously been described in wine and whether they have a similarly detrimental role is unknown. These results suggest that inoculation not only expedites fermentation via introduction of a healthy, active strain of *Saccharomyces*, but also via limitation of bacterial diversity, in turn checking the potential for problem fermentations related to bacterial growth [38]. This conclusion confirms empirical praxis underlying the growing practice of inoculation among winemakers globally. Further study of how the rare bacterial taxa of wine fermentation interact with inocula to impact fermentation kinetics warrants additional attention. Aided by the extreme sensitivity and phylogenetic diversity metrics available with NGS tools, such studies probing the microbial “terroir" of wine and its impact on fermentation performance and wine quality are now possible.

## Supporting Information

Figure S1 Observed species (left) and PD Whole Tree (right) Alpha Rarefaction of BAS sequences by Sample. A,B: V4 5′. C,D: V4 3′. E,F: V5 4′. G,H: V5 3′.
(TIFF)

Figure S2 Taxonomic key for Figure 2.
(TIFF)

Table S1 V4 primers and barcodes used in this study.
(DOC)

Table S2 V5 barcodes used in this study.
(DOC)

Table S3 Sequencing Raw Read and Quality Filtration Statistics.
(XLS)

Table S4 BAS genus-level taxonomy for V4 5′ reads.
(XLS)

Table S5 BAS genus-level taxonomy for V4 3′ reads.
(XLS)

Table S6 BAS genus-level taxonomy for V5 5′ reads.
(XLS)

Table S7 BAS genus-level taxonomy for V5 3′ reads.
(XLS)
