# Correction: Next-Generation Sequencing Reveals Significant Bacterial Diversity of Botrytized Wine

**DOI:** 10.1371/annotation/4d347090-bca1-4184-a5d5-bc2d38baec7d

**Published:** 2013-10-08

**Authors:** Nicholas A. Bokulich, C. M. Lucy Joseph, Greg Allen, Andrew K. Benson, David A. Mills

In the first sentence of the “Library Construction” section the sequence for primer F515 appears incorrectly in the text. The sentence should read: “For amplification of the V4 domain of bacterial 16S rDNA, we used primers F515 (5′-GTGCCAGCMGCCGCGGTAA-3′)”

