# Supplementary figures and images for: Next-Generation Sequencing Reveals Significant Bacterial Diversity of Botrytized Wine
Source: PLoS One. 2012 May 1;7(5):e36357. doi: 10.1371/journal.pone.0036357 (PMC3341366; doi:10.1371/journal.pone.0036357)

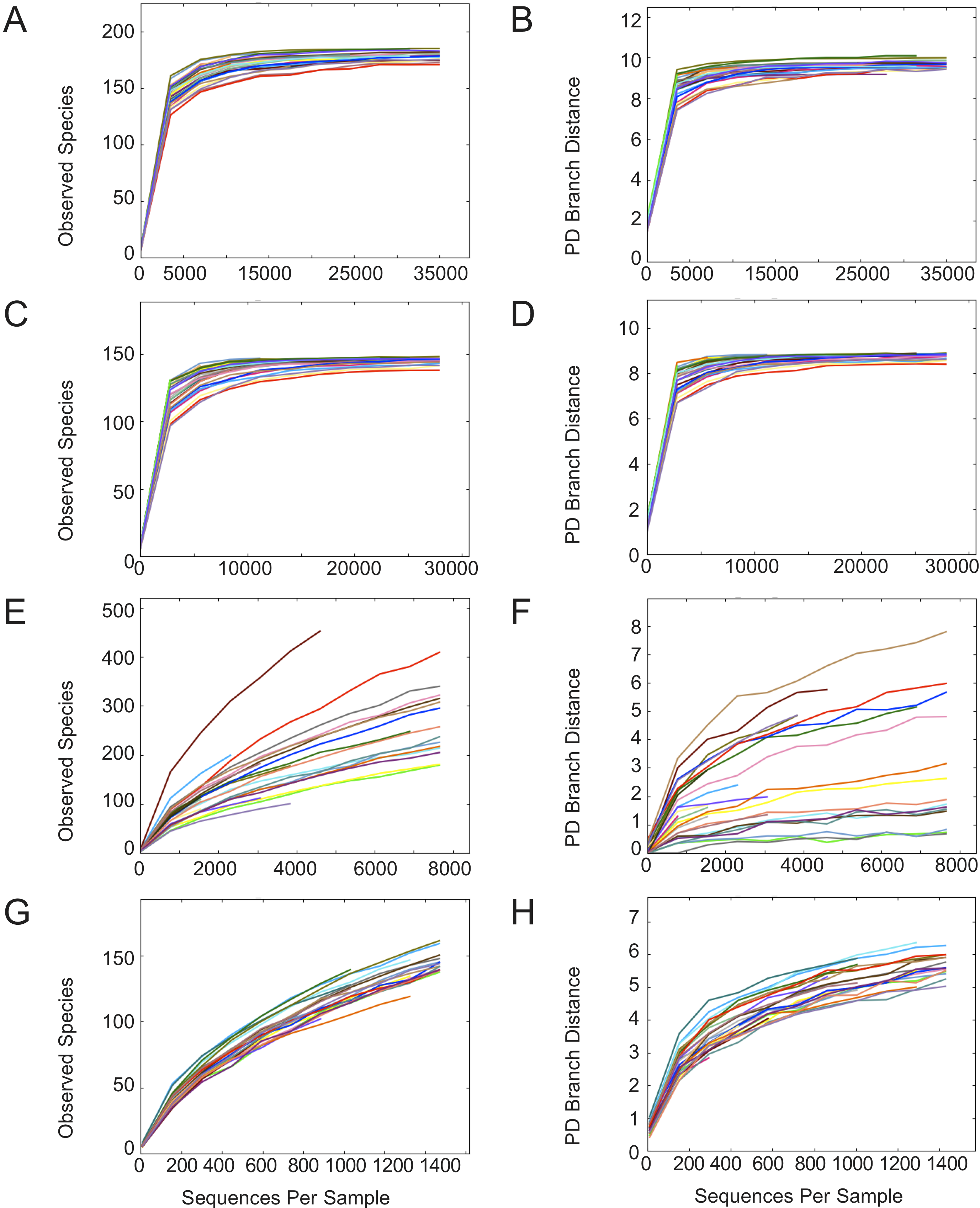

Supplement: Figure S1 — Observed species (left) and PD Whole Tree (right) Alpha Rarefaction of BAS sequences by Sample. A,B: V4 5′. C,D: V4 3′. E,F: V5 4′. G,H: V5 3′. (TIFF) [file pone.0036357.s001.tiff]

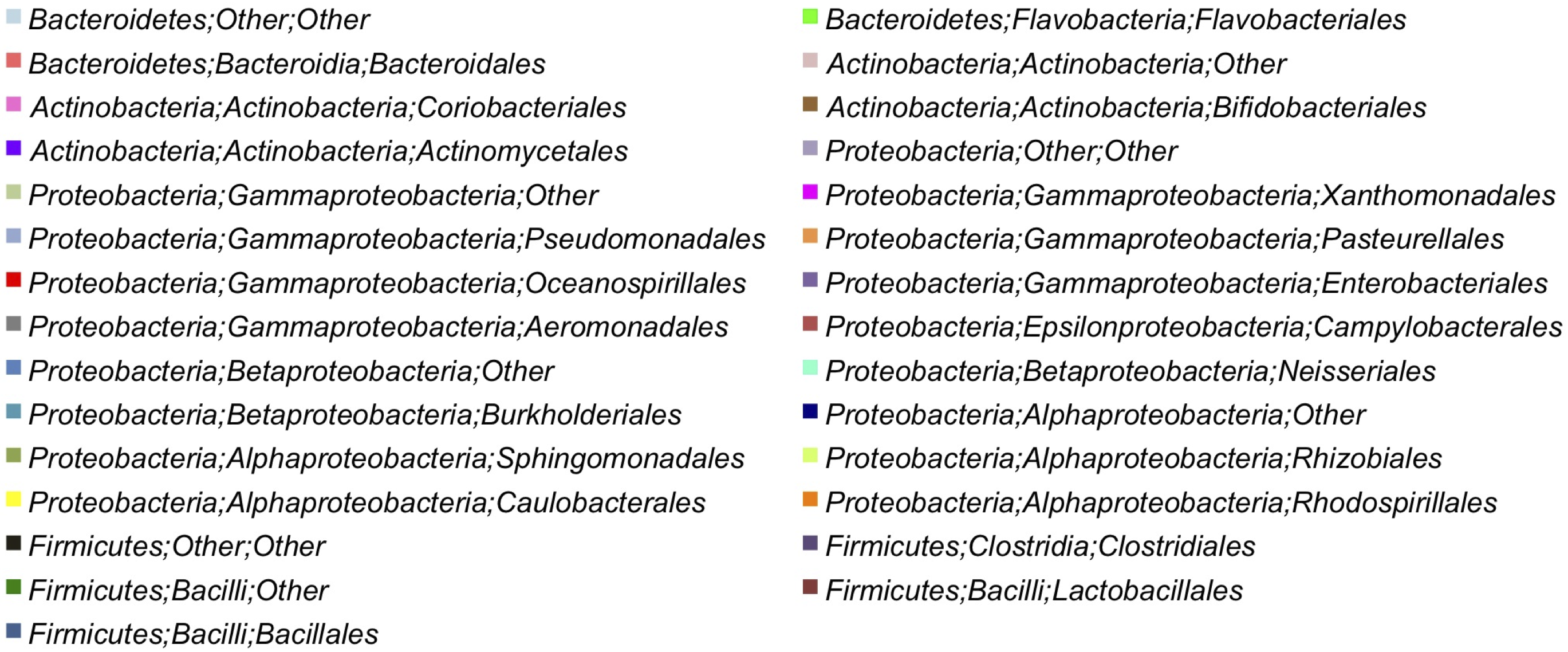

Supplement: Figure S2 — Taxonomic key for Figure 2. (TIFF) [file pone.0036357.s002.tiff]
