# Supplementary material for: Next-Generation Sequencing Reveals Significant Bacterial Diversity of Botrytized Wine
Source: PLoS One. 2012 May 1;7(5):e36357. doi: 10.1371/journal.pone.0036357 (PMC3341366; doi:10.1371/journal.pone.0036357)
Supplement: Table S1 — V4 primers and barcodes used in this study. (DOC) [file pone.0036357.s003.doc]

**Table S1.** V4 primers and barcodes used in this study.

Underlined region indicates Illumina adapter sequence. Bold-face text indicates PCR primer region, preceded by linker sequence. Poly-N string in forward primer denote barcode sequence.

Forward Primer:

AATGATACGGCGACCACCGAGATCTACACTCTTTCCCTACACGACGCTCTTCCGATCTNNNNNNNNGT**GTGCCAGCMGCCGCGGTAA**

Reverse Primer:

CAAGCAGAAGACGGCATACGAGATCGGTCTCGGCATTCCTGCTGAACCGCTCTTCCGATCTCC**GGACTACHVGGGTWTCTAAT**

Barcodes used in this study:

AACCAACC

AACCAAGG

AACCATCG

AACCATGC

AACCGCAT

AACCGCTA

AACCGGAA

AACCGGTT

AACCTACG

AACCTAGC

AACCTTCC

AACCTTGG

AACGAACG

AACGAAGC

AACGATCC

AACGATGG

AACGCCAT

AACGCCTA

AACGCGAA

AACGCGTT

AACGGCAA

AACGGCTT

AACGTACC

AACGTAGG

AACGTTCG

AACGTTGC

AAGCAACG
