# Supplementary material for: Next-Generation Sequencing Reveals Significant Bacterial Diversity of Botrytized Wine
Source: PLoS One. 2012 May 1;7(5):e36357. doi: 10.1371/journal.pone.0036357 (PMC3341366; doi:10.1371/journal.pone.0036357)
Supplement: Table S2 — V5 barcodes used in this study. (DOC) [file pone.0036357.s004.doc]

**TABLE S2:** V5 barcodes used in this study.

ATCACG

TTAGGC

TGACCA

ACAGTG

GCCAAT

CAGATC

ACTTGA

GATCAG

TAGCTT

GGCTAC

CTTGTA

GGTTAA

CCAGGA

GGCAGT

AAGACT

GGGTTG

TTTCCT

CCTCGA

CCACCA

AAATTA

CCCAAC

TTGCAT

CCCGGC

TTGTCA

AAAGTC

TTCACA

CCGTGT

GGAGCT
